# Metformin Promotes Axonal Regeneration and Functional Recovery in Diabetic Rat Model of Sciatic Nerve Transection Injury

**DOI:** 10.3390/neurosci3030026

**Published:** 2022-06-21

**Authors:** Junxiong Ma, Jun Liu, Yu Chen, Hailong Yu, Liangbi Xiang

**Affiliations:** Department of Orthopedics, General Hospital of Northern Theater Command, Shenyang 110000, China; majx0221@163.com (J.M.); liujun0355@163.com (J.L.); chenyu0232@sina.com (Y.C.)

**Keywords:** metformin, nerve regeneration, functional recovery, sciatic nerve injury, neuroprotective

## Abstract

In our previous study, metformin was able to promote nerve regeneration after sciatic nerve crushing in rats under diabetic conditions. However, a crush injury also has a strong ability to spontaneously recover. Therefore, in our present study, a model of transection injury of the sciatic nerve in diabetic rats was utilized to detect whether metformin could still promote nerve regeneration. Diabetes was induced via an injection of 50 mg/kg of streptozotocin in rats. After transection injury of the sciatic nerve, the rats were randomly divided into a high-dose metformin group (500 mg/kg/d), mid-dose metformin group (200 mg/kg/d), low-dose metformin group (30 mg/kg/d) and control group (normal saline). The metformin or normal saline was intraperitoneally injected for 4 weeks. Then, behavioral, electrophysiological and morphometric analyses were performed. The results showed that metformin could significantly promote functional restoration and axonal regeneration of the sciatic nerve after transection injury under diabetic conditions. Furthermore, high doses and middle doses of metformin presented more of this ability than a low dose of metformin. In conclusion, metformin is able to accelerate sciatic nerve repair after transection injury under diabetic conditions, showing the therapeutic potential of metformin in the management of nerve injuries during diabetes mellitus.

## 1. Introduction

As one of the most common and important global health issues, it is estimated that diabetes mellitus will affect more than 500 million people by 2030 [1]. Diabetes can lead to a variety of complications, of which peripheral diabetic neuropathy is the most common, leading to permanent disability via the gradual loss of nerve fibers and atrophy of axons [2]. In addition, the capacity of neural regeneration is dramatically impaired in diabetic animal models of peripheral nerve injury, which generally leads to disability [3]. Thus far, it has been a big challenge to treat peripheral nerve injury under diabetic condition. 

Metformin is a classic drug for diabetes mellitus. It possesses a series of pharmacological properties, such as anti-tumor, anti-oxidant and anti-inflammatory abilities [4,5,6,7]. In recent studies, it has been recognized that metformin possesses neuroprotective effects on the nervous system. Metformin was found to reduce neurogenic pain induced by chemotherapy in a mouse model [6]. In addition, metformin has been shown to increase apolipoprotein E expression. As apolipoprotein E is the key factor in neural regeneration, metformin might play an important role during nerve repair [7]. In our previous study, metformin showed beneficial effects on painful diabetic neuropathy in rats [8]. In our previous study, metformin is also capable of promoting restoration of nerve function after a crush injury of the sciatic nerve in rats under diabetic conditions [9]. In a recent study, metformin was also proved to be able to promote nerve repair after a crush injury in rats under non-diabetic conditions [10]. Despite the findings, it should be noted that a crush injury is not considered very serious damage to the neural tissue, which has a strong ability for spontaneous recovery. The promoting effect of metformin on nerve repair under more serious conditions of nerve injury, such as a transection injury or lengthy nerve defects, under diabetic conditions, is still not clear. A model of a transection injury of the sciatic nerves in diabetic rats was used in our present study to detect the therapeutic potential of metformin for nerve repair and functional recovery under diabetic conditions.

## 2. Materials and Methods

### 2.1. Diabetic Rat Model of Sciatic Nerve Transection Injury

All experiments with animal were performed in line with the guidelines of the China Council on Animal Care and Use. This study obtained approval by the Committee of Experimental Animals of General Hospital of Northern Theater Command (approval number: GK20190929) on 29 September 2019. Adult male Sprague–Dawley rats, weighing from 200 g to 240 g, were provided by the Laboratory Animal Center of the General Hospital of Northern Theater Command. Diabetes (*n* = 120) in rats was induced through intraperitoneally administration of 50 mg/kg of streptozotocin (STZ). Blood glucose levels were measured before noon twice a week from tail vein samples using a commercially available glucometer (Freestyle, Abbott Diabetes Care, Inc.; Alameda, CA, USA). The blood glucose was 15.24 ± 1.35 mmol/L at 3 weeks after STZ administration, which is more than 13.89 mmol/L, indicating the establishment of diabetes. On the 21st day after STZ injection, 40 mg/kg of sodium pentobarbital solution was intraperitoneally injected for use for the anesthesia of the diabetic rats. An incision was made in the left thigh skin and was followed by an incision made directly through the biceps femoris muscle, exposing the sciatic nerve and its three terminal branches (the sural, common peroneal, and tibial nerves). The tibial and common peroneal branches of the sciatic nerve were transected distally, while the sural nerve was left intact. Proximal and distal ends of nerve stumps were sutured by three perineural 10/0 nylon sutures in 96 rats. The remaining rats served as a sham-surgery group which were subjected to sciatic nerve exposure, but no transection injury.

### 2.2. Experimental Groupings

After transection injury of sciatic nerve, the rats were randomly divided into a high-dose metformin group (500 mg/kg/d, *n* = 24), mid-dose metformin group (200 mg/kg/d, *n* = 24), low-dose metformin group (30 mg/kg/d, *n* = 24) and vehicle group (normal saline, *n* = 24). The metformin (Sigma, St. Louis, MO, USA) or normal saline was intraperitoneally injected for 4 weeks. Furthermore, metformin was dissolved in the same volume of saline which was injected in the vehicle group.

### 2.3. Sciatic Functional Index (SFI)

SFI was calculated on all the rats at 4, 8 and 12 weeks after injury. Before anesthesia, non-toxic finger paint was used to paint the hind paws of the rats [11]. Afterwards, the rats were divided and ran into a dark target box through a 50 × 7 cm wooden pathway. The footprints were gathered to calculate SFI using the formula below:

SFI = (−38.3 × (EPL − NPL)/NPL) + (109.5 × (ETS − NTS)/NTS) + (13.3 × (EIT − NIT)/NIT) − 8.8

In this formula, PL, the abbreviation for “print length”, means the distance between the 3rd toe tip and the heel; IT, the abbreviation for “intermediary toe spread”, means the distance from the 2nd toe to the 4th toe; TS, the abbreviation for “toe spread”, means the distance between the 1st toe and 5th toe. In addition, E means the distance measured from the operation side of the paw, and N means the distance measured from the contralateral side of the paw. For example, EPL means the PL measured from the operation side of the paw.

### 2.4. Thermal Sensation Recovery by Hot Plate Test

Twenty-four h after the measurement of SFI, thermal sensory function was detected via a hot plate test [12]. Briefly, the rats were kept in an independent cage separately for one day. Then, the rats were made to stand with a 56 °C hot plate under the paw of operation side. Radiant heat was used to heat hot plates. The latency time until the rats withdrew the paw was recorded. When the rats did not withdraw the paw until 20 s, the test was stopped in order to avoid heat injury. The measurement was repeated four times with a 5 min inter-stimulus interval.

### 2.5. Mechanical Sensory Recovery

Twenty-four h after the measurement of thermal sensory function, the mechanical sensory function was detected according to mechanical withdrawal threshold. The von Frey filament with incremental forces (from 0.38 g to 15.1 g) was used to test the mechanical sensitivity. Briefly, a special cage with wire-mesh-bottom was prepared. After placing the rats in the cage, the filament was used to stick the hind paw for 3 to 5 s in an ascending order. A 30 s interval was left between stimulations of different forces. We were careful to avoid continuous stimulation of the same location of the foot pads. The positive response was defined as abrupt withdrawal and licking or shaking of the paw. The minimum force evoking positive responses at least five times was considered as the mechanical withdrawal threshold [13].

### 2.6. Electrophysiological Recording and Analysis

Motor function was detected according to electrophysiological test 24 h after the measurement of mechanical sensory function. Briefly, the exposure of the left-side sciatic nerve was finished under anesthesia. The transection site was identified, and the stimulating electrode was put under the nerve proximally to the transection site, with a distance of 10 mm. Compound muscle action potentials (CMAPs) was recorded through recording electrodes placed in the gastrocnemius muscle. The electrophysiological test was performed in the contralateral side in the same way. The calculations of nerve conduction velocity (NCV), CMAP latency of onset and CMAP peak amplitude were performed according to a method described previously [14].

### 2.7. Fluoro-Gold Retrograde Labeling

After the electrophysiological test, retrograde labeling of Fluoro-Gold (FG) was carried out. The exposure of the left sciatic nerve was finished under anesthesia. FG injection (4%, 2 μL) was performed on the nerve 5 mm distal to the suture site using a 2 μL glass micropipette. The FG was purchased from Biotium Inc., Frisco, CA, USA. Then, the wound was closed again. Next, 72 h later, the animals were sacrificed through transcarotid perfusion with paraformaldehyde (4%, *w*/*v*), which was dissolved in phosphate buffer (0.1 M). Then, the spinal cords and dorsal root ganglions (DRGs) at the levels of L4, L5 and L6 were harvested. The sample was post-fixed for 12 h in 4% paraformaldehyde buffer, and immersed in 30% sucrose with anti-freezing agent at 4 °C overnight. The 25 µm-thick transverse sections were prepared on a cryostat. The FG-labeled motoneurons in the spinal cords as well as FG-labeled sensory neurons in DRGs were counted as previously reported [15]. Briefly, sections were serially mounted on glass slides, dried, and coverslipped. Each spinal cord section was visualized under UV fluorescence by an observer who was unaware of which branch had received FG or FB. Motoneurons containing both FG and FB throughout the cell body were viewed by changing the fluorescent light. The counting of split cells twice was corrected for by the method of Abercrombie and colleagues (1946). In each group, motoneurons were scored as projecting axons (1) correctly to the muscle branch, (2) incorrectly to the cutaneous branch, or (3) simultaneously to both branches.

### 2.8. Morphometric Analysis

Next, 4, 8 and 12 weeks after injury, the sciatic nerve 2 mm anterior and posterior to the transected site was obtained. Then, the samples were fixed in glutaraldehyde (3%). The nerves were post-fixed in osmium tetroxide (1%) in pH 7.3 sodium cacodylate buffer (0.1 M). After being dehydrated in ethanol, the samples were embedded in resin. Then, 1.0 μm-thick semi-thin sections and 50 nm-thick ultra-thin sections were prepared for further observation. AH3 light microscope (Olympus, Tokyo, Japan) was used to examine the toluidine blue/borax-stained semi-thin sections. H-600 transmission electron microscope (HITACHI, Tokyo, Japan) was employed to examine the ultra-thin sections, which were stained through uranyl acetate/lead citrate. The nerve fiber diameter and the axon myelination were measured and calculated for the estimation of axonal regeneration [16]. Briefly, morphometric evaluations were conducted by examiners who were blinded to the experimental design. In each group, axonal regeneration was estimated by (1) the total number of myelinated axons per nerve transverse section (Mtot) (measurement by imageJ), (2) the total area of regenerated nerves (Atot) (measurement by imageJ), and (3) the mean diameter of nerve fibers (measurement by imageJ). The degree of myelination was estimated by the axon-to-fiber diameter ratio (G-ratio).

### 2.9. Statistical Analysis

All the data are expressed as the means ± standard errors of the means (SEMs). The mean values were compared through one-way analysis of variance (ANOVA) using the SPSS 13.0 software package (SPSS Inc., Chicago, IL, USA). If there was a significant overall difference among groups, Tukey’s post hoc test was used to make pair-wise comparisons. Values of *p* < 0.05 were considered statistically significant.

## 3. Results

### 3.1. Enhanced Axonal Regeneration by Metformin in Diabetic Rats

**Figure 1:**
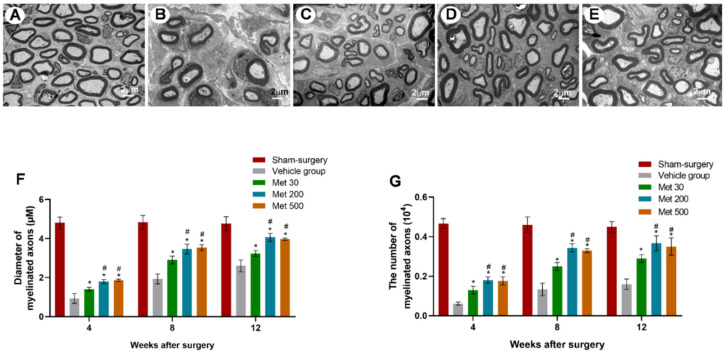
Representative micrographs of transection site in sciatic nerve under transmission electron microscopy (**A**–**E**) in the sham-surgery group (**A**), vehicle group (**B**), and groups with metformin at 30 mg/kg (**C**), 200 mg/kg (**D**), and 500 mg/kg (**E**) at 12 weeks after injury. The diameter of myelinated axons (**F**) and the number of myelinated axons (**G**) were counted and expressed as the mean ± standard error of mean. * *p* < 0.05 for comparison to the vehicle group. # *p* < 0.05 for comparison to rats with metformin at low dose (30 mg/kg).

In diabetic rats, enhanced axonal regeneration could be seen in metformin group after transection injury of the sciatic nerve. The axon diameter as well as axon myelination in metformin groups were markedly better than those in the vehicle group after injury (4, 8 and 12 w) (*p* < 0.05, Figure 1). The results indicated that metformin could promote axonal regeneration in diabetic conditions after a transection injury. Furthermore, the axon diameter and axon myelination in high-dose and mid-dose metformin groups were similar, but much higher than those in low-dose metformin group, indicating that metformin could promote axonal regeneration in a dose-dependent manner under diabetic conditions (*p* < 0.05, Figure 1).

**Figure 2:**
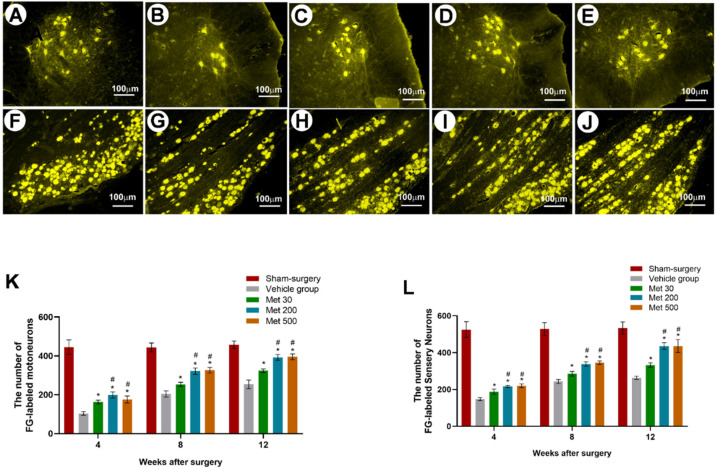
Representative micrographs of FG-labeled motoneurons (**A**–**E**) and sensory neurons (**F**–**J**) (L4–L6) in the sham-surgery (**A**,**F**), vehicle group (**B**,**G**), metformin at 30 mg/kg (**C**,**H**), 200 mg/kg (**D**,**I**), and 500 mg/kg (**E**,**J**) at 12 weeks after injury. The number of FG-positive motor neurons (**K**) and sensory neurons (**L**). All data are expressed as the mean ± standard error of mean. * *p* < 0.05 for comparison to the vehicle group. # *p* < 0.05 for comparison to rats with metformin at low dose (30 mg/kg).

Retrograde labeling of FG was conducted to identify whether the neurons regenerated through the injury site and arrived at the distal part of the nerve. The FG-positive sensory or motor neurons of metformin groups were much more abundant than those of the vehicle group after injury (4, 8 and 12 w) (*p* < 0.05, Figure 2). Furthermore, the FG-positive sensory or motor neurons of high-dose and mid-dose metformin groups were similar, but much more abundant than those of the low-dose metformin group (*p* < 0.05, Figure 2). These results indicate that metformin is able to promote neuron regeneration through the injury site into the DRG in a dose-dependent manner.

### 3.2. Metformin Promotes Recovery of Motor Function

**Figure 3:**
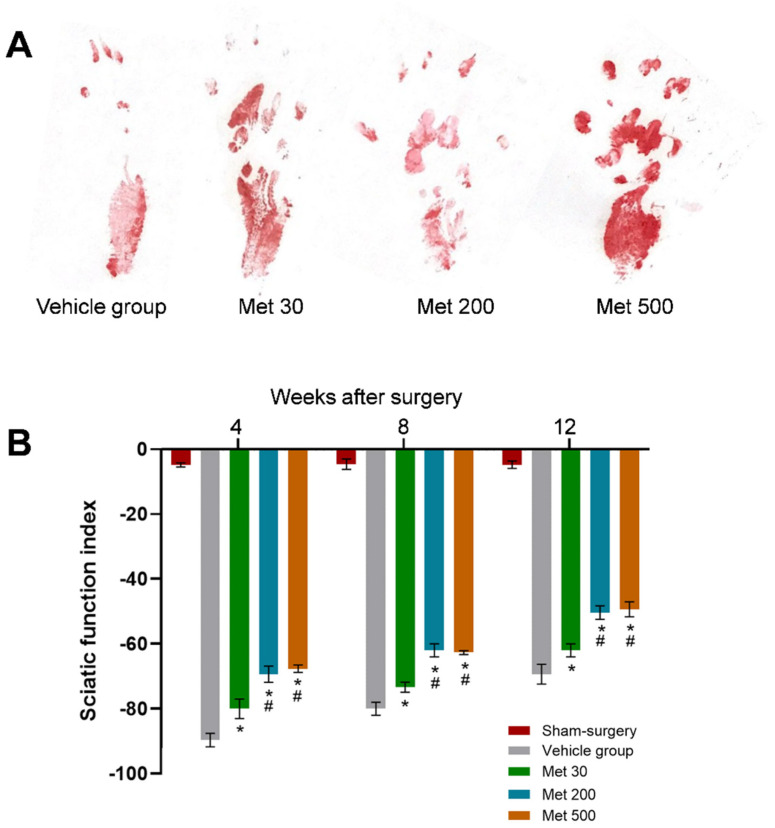
The SFI in each group. (**A**) Representative images of operative left footprints 12 weeks following the operation; (**B**) SFI values 4, 8 and 12 weeks following the operation. All data are expressed as the mean ± standard error of mean. * *p* < 0.05 for comparison to the vehicle group. # *p* < 0.05 for comparison to rats with metformin at low dose (30 mg/kg).

One of the primary indices for evaluating sciatic nerve function is gait analysis. SFI values of metformin groups were dramatically better than in the vehicle group (*p* < 0.05, Figure 3A,B). Furthermore, the SFI values were similar between high-dose and mid-dose metformin groups, but much better than those of the low-dose metformin group (*p* < 0.05, Figure 3A,B). These results indicate that metformin could promote the recovery of motor function in a dose-dependent manner after the transection injury of the sciatic nerve under diabetic conditions.

**Figure 4:**
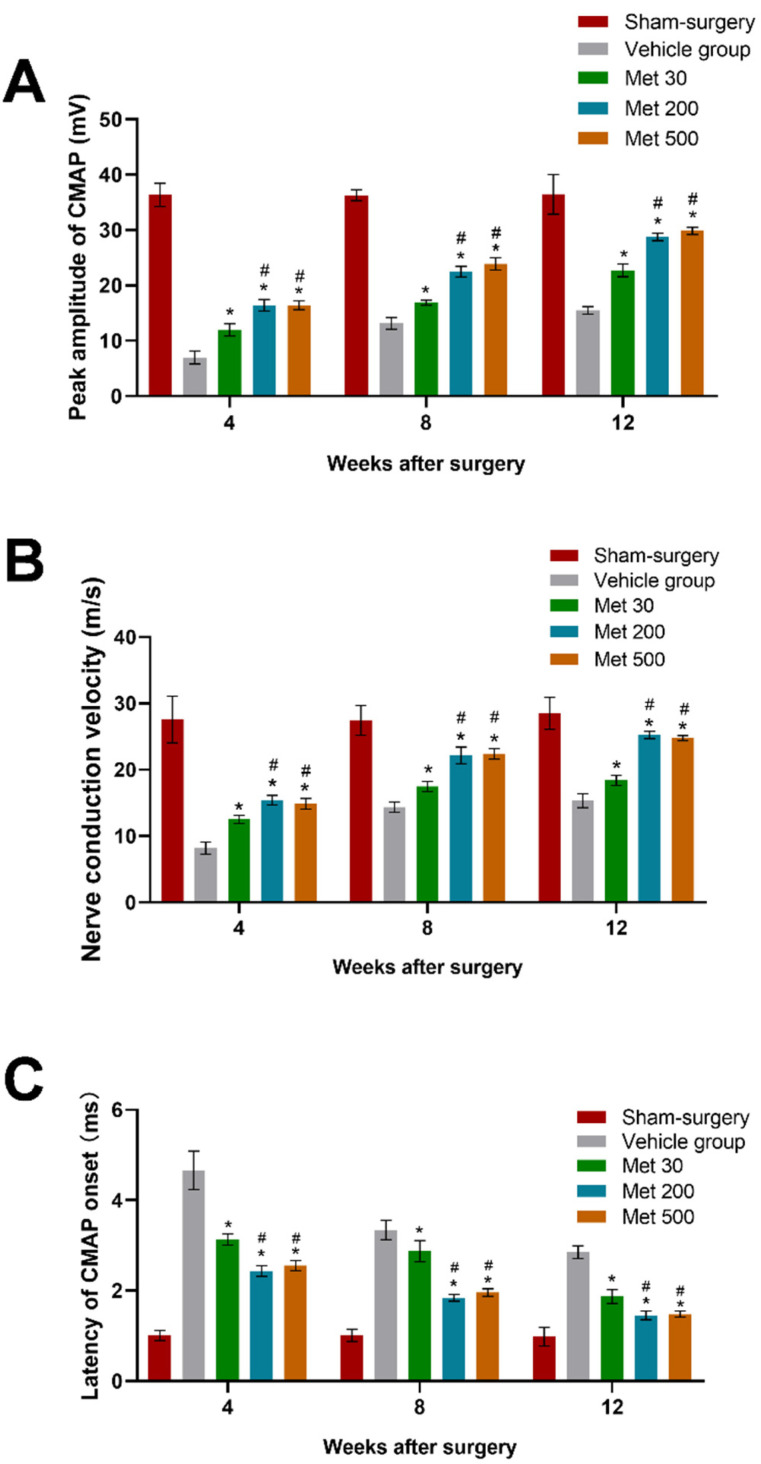
The result of electrophysiological tests at each point in time in each group. The peak amplitude of CMAP (**A**), nerve conduction velocity (**B**) and latency of CMAP (**C**) were recorded and expressed as the mean ± standard error of mean. * *p* < 0.05 for comparison to the vehicle group. # *p* < 0.05 for comparison to rats with metformin at low dose (30 mg/kg).

Electrophysiological analysis was used to further detect the effect of metformin on the recovery of motor function of diabetic rats after sciatic transection. Compared with the vehicle group, metformin groups showed better NCV, CMAP latency of onset and CMAP amplitude (*p* < 0.05, Figure 4), indicating the beneficial effect of metformin on recovery of motor function. Furthermore, NCV, CMAP latency of onset and CMAP amplitude in high-dose and mid-dose metformin groups were similar, and markedly better than in the low-dose metformin group (*p* < 0.05, Figure 4). These findings suggest that metformin can dose-dependently enhance the recovery of motor function.

### 3.3. Metformin Promotes Recovery of Sensory Function in Diabetic Rats

**Figure 5:**
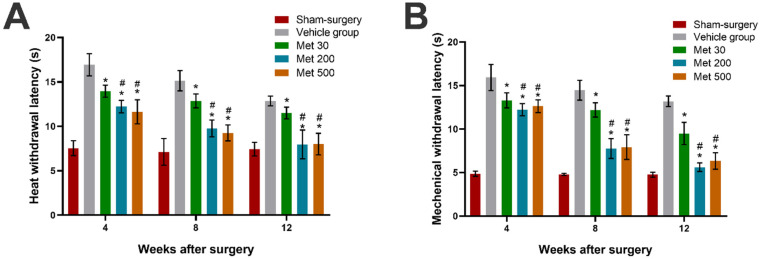
The thermal (**A**) and mechanical (**B**) withdrawal thresholds at each time point in each group. All data are expressed as the mean ± standard error of mean. * *p* < 0.05 for comparison to the vehicle group. # *p* < 0.05 for comparison to rats with metformin at low dose (30 mg/kg).

One of the primary indices for evaluating sensory nerve function is thermal sensation analysis, which examined by the hot plate test in the present study. Metformin treatment significantly reduced the withdrawal latency to heat stimuli (*p* < 0.05, Figure 5A), suggesting that metformin plays a key role in enhancing recovery of heat sensation of diabetic rats with transection injury of the sciatic nerve. Furthermore, the heat withdrawal latency of the high-dose and mid-dose metformin groups was similar, and markedly better than in the low-dose metformin group. These results indicate that metformin can dose-dependently enhance the recovery of sensory function (*p* < 0.05, Figure 5A).

The recovery of mechanical sensation was examined using von Frey filaments. After injury, metformin treatment significantly lowered the mechanical withdrawal threshold compared to the vehicle treatment (*p* < 0.05, Figure 5B), suggesting that metformin can promote mechanical sensory recovery under diabetic conditions after sciatic transection. Furthermore, a dose-dependent effect on mechanical sensation recovery was found in metformin. The mechanical withdrawal thresholds were similar between the high-dose and mid-dose metformin groups, and were much better than in the low-dose metformin group (*p* < 0.05, Figure 5B).

## 4. Discussion

Our present study validated whether metformin could promote axonal regeneration after transection injury of the peripheral nerve in diabetic rats. The results showed that metformin possesses the capability of enhancing axonal repair and promoting the recovery of both motor and sensory function after sciatic transection in diabetic rats. Furthermore, the regeneration of axons and recovery of nerve function of high-dose and mid-dose metformin groups were similar, and much better than in the low-dose metformin group. All these results illustrate the potential of metformin for management of nerve injuries under diabetic conditions.

Metformin and its neural protective function have been investigated under both diabetic condition and normal conditions [4,5,6,7]. It was proved that diabetes mellitus might impair the capability of neural regeneration. In our previous study, metformin could promote axonal repair and the recovery of nerve function after a crush injury of the sciatic nerve in rats. However, a crush injury of the sciatic nerve is not considered very serious damage to the neural tissue, which has a strong ability to spontaneously recover. Therefore, the present study studied whether metformin could promote axonal repair in a more severe injury model (transection injury). The results showed that axon myelination and FG-positive neurons in the metformin groups were extremely better than in the vehicle, revealing that metformin can promote regeneration of axons after transection injury of the sciatic nerve under diabetic conditions. This may be the case. However, Diamond found that the sprouting of intact cutaneous nerves innervating adjacent skin regions may play a role in recovery of the cutaneous sensation of denervated skin [16]. Furthermore, it was reported that collateral sprouting of intact neighboring axons has been shown to significantly contribute to reinnervation of denervated skin following lesions of hindlimb nerves in rats [17,18]. Hence, further studies are required to determine the mechanism by which metformin promotes axonal regeneration.

Axonal regeneration is not equal to functional recovery, because the function will not recover until the axons regenerate through the transection site and arrive at the distal targets. An electrophysiological test was conducted to evaluate the effect of metformin on motor function recovery, since CMAP amplitude has been found to be related to the number of regenerative axons arriving at the target muscle [19,20]. The results showed that metformin treatment significantly increased NCV and CMAP amplitude while reducing CMAP latency, illustrating the effect of metformin on promoting recovery of motor function in diabetic rats. In addition, high-dose and mid-dose metformin groups showed better NCV, CAMP peak amplitude and CAMP latency than the low-dose group, suggesting metformin might promote functional recovery in a dose-dependent manner. Further behavioral studies showed that metformin could achieve a better SFI, suggesting that it could promote recovery of motor function. Besides recovery of motor function, a dose-dependent promoting effect of metformin on sensation recovery was observed in diabetic rats with sciatic transection injury, which was evidenced by observations in which metformin significantly decreased both the heat withdrawal latency and mechanical withdrawal threshold. The findings of our study suggest that metformin can enhance the recovery of nerve functions after transection injury of the sciatic nerve in diabetic rats.

The maximal clinical dosage of metformin is 2.5 g/day for adults, which could be calculated as 35 mg/kg for a body weight of 75 kg [21]. Although high doses (500 mg/kg) and middle doses (200 mg/kg) of metformin showed a stronger ability to promote regeneration of axons and recovery of nerve function than a low dose (30 mg/kg) of metformin, the low dose (30 mg/kg) may hold better clinical significance because it approached the highest dose in clinical application. In addition, the present results indicated the effect of metformin on sciatic transection injury in diabetic rats.

Metformin is a widely prescribed drug used in the treatment of type II diabetes. While the drug has many mechanisms of action, most of these converge on AMP-activated protein kinase (AMPK), which metformin activates. AMPK is a multifunctional kinase that is a negative regulator of mechanistic target of rapamycin (mTOR) and mitogen-activated protein kinase (MAPK) signaling. Activation of AMPK decreases the excitability of dorsal root ganglion neurons, and AMPK activators are effective in reducing chronic pain in inflammatory, post-surgical and neuropathic rodent models. Previous research has shown that metformin leads to an enduring resolution of neuropathic pain in a spared nerve injury (SNI) model in male mice and rats. The underlying molecular mechanism responsible for its neuroprotective action is still unknown. This is a limitation of our present study. We hypothesized that metformin attenuates sciatic nerve injury in diabetic rats by suppressing inflammation, oxidative stress and fibrosis. Further investigation should be undertaken in the future to identify the possible mechanism for this.

In conclusion, metformin promoted regeneration of axons and recovery of nerve function after transection injury of the sciatic nerve in diabetic rats. High doses (500 mg/kg) and middle doses (200 mg/kg) of metformin achieved better effects than low doses (30 mg/kg). The results of our study indicate the therapeutic potential of metformin for nerve injuries under diabetic conditions.

## Data Availability

The data used and/or analyzed during the current study are available from the corresponding author upon reasonable request.
